# Deflection-based laser sensing platform for selective and sensitive detection of H_2_S using plasmonic nanostructures

**DOI:** 10.1038/s41598-022-19739-8

**Published:** 2022-09-22

**Authors:** Elham Afjeh-Dana, Elham Asadian, Mohammad Reza Razzaghi, Hashem Rafii-Tabar, Pezhman Sasanpour

**Affiliations:** 1 Department of Medical Physics & Biomedical Engineering, School of Medicine, Shahid Beheshti University of Medical Sciences, Tehran, Iran; 2 Laser Application in Medical Sciences Research Center, Shahid Beheshti University of Medical Sciences, Tehran, Iran; 3 The Physics Branch of Iran Academy of Sciences, Tehran, Iran; 4 School of Nanoscience, Institute for Research in Fundamental Sciences (IPM), P. O. Box 19395-5531, Tehran, Iran

**Keywords:** Imaging and sensing, Optical metrology, Nanophotonics and plasmonics, Sensors, Computational science

## Abstract

Considering the severe hazards of abnormal concentration level of H_2_S as an extremely toxic gas to the human body and due to the disability of olfactory system in sensing toxic level of H_2_S concentration, a reliable, sensitive, selective and rapid method for the detection of H_2_S is proposed and its efficacy is analyzed through simulation. The proposed system is based on the deflection of a laser beam in response to the temperature variations in its path. In order to provide selectivity and improve sensitivity, gold nanostructures were employed in the system. The selectivity was introduced based on the thiol–gold interactions and the sensitivity of the system was enhanced due to the modification of plasmon resonance behavior of gold nanostructures in response to gas adsorption. Results from our analysis demonstrate that compared with Au and SiO_2_–Au, the Au nanomatryoshka structures (Au–SiO_2_–Au) showed the highest sensitivity due to promoting higher deflections of the laser beam.

## Introduction

Hydrogen sulfide (H_2_S), is a colorless water-soluble, corrosive, flammable and extremely toxic gas and is identified with a “rotten egg” odor. H_2_S is widely produced in nature or industry, such as in hot springs, volcanic gases, crude petroleum, petrochemical industry, paper manufacturing, and waste disposal^1–5^. Many investigations have demonstrated that H_2_S in abnormal concentration levels, has serious adverse effects on human health. Numerous neural disorders, such as ischemic stroke, Alzheimer’s disease, Parkinson’s disease, Down’s syndrome, could occur because of abnormal levels of H_2_S^2,3,6^. Also, H_2_S could affect the cardiovascular system due to the opening of the ATP-sensitive potassium channel, leading to vascular smooth muscle relaxation and a decrease in blood pressure^3^. Furthermore, H_2_S can highly affect the eyes, skin, the respiratory system, and mucous membranes could be destroyed or inflamed^7,8^. H_2_S with concentrations higher than 250 ppm could lead to blood poisoning and even death^1^. In this regard, considering the human and environmental safety, the safe exposure threshold of H_2_S announced by the American National Institute for Occupational Safety and Health (NIOSH) is 10 ppm for 8 h^9^.

The olfactory organs of humans can feel H_2_S in concentration of 130 ppb with a characteristic similar to a rotten egg smell, while at the concentration of 83 ppb it interacts with blood hemoglobin with destructive effects on human health^5^. In addition, small increase in H_2_S levels, or prolonged exposure to low concentrations, can cause anosmia^3^. Therefore, the design and fabrication of a rapid and reliable sensing platform for in-situ real-time detection of H_2_S in ppm concentration with high selectivity and sensitivity is a major challenge^1,3,8^.

So far, many strategies have been developed for detection of H_2_S which could be classified under three main categories; semiconductor metal oxide (SMO) (such as ZnO, SnO_2_, In_2_O_3_)^10^, electrochemical^11^ and optical based sensors^3,12^. Among various types of optical-based sensors, fluorescence-based detection^13^, colorimetry^14^, surface enhanced Raman spectroscopy (SRES)^15^, and UV–visible absorption spectrometry^16^ are well known. Despite advances in H_2_S detection in the past few years, these techniques have suffered from certain limitations. For instance, in mobile monitoring of H_2_S by SMO-based sensors, the main limitation is the power consumption^17^. In case of the electrochemical sensors, the impact of ambient humidity and temperature is a potential limitation^18^. Although electrochemical-based sensors are able to overcome the limitation of temperature and humidity dependence to some extent, but high temperatures interfere with the performance of these sensors^19^. Despite the high sensitivity and selectivity of fluorescence-based sensor, difficulty in the synthesis of tags and their durability, restricts its application^6^. Moreover, colorimetry-based detection techniques lack sufficient sensitivity for H_2_S gas^2^.

Considering these limitations of the current methods, design of novel strategies for detection of H_2_S with the capability of overcoming the above restrictions seems necessary. Non-contact optical-based sensors are not affected by temperature or humidity and maintain their functionality even in high temperature and ambient humidity^1^. As one of the most sensitive members of molecular absorption spectroscopy family, beam deflection spectroscopy (BDS) can be utilized for detecting agents as well as the measurement of special sample properties such as porosity and thermal properties^20–23^. It is also known as “photothermal deflection spectroscopy (PDS)” or “mirage-effect technique”^20,24,25^. Briefly, in the BDS, the sample is locally irradiated using a modulated laser beam (pump beam), and the absorbed electromagnetic radiation heats the sample locally through non-radiative processes. The refractive index of medium would change as a result of variations in the density and consequently, the path of another laser beam (probe beam) which passes along the sample surface, would be spatially deflected^24,26^. By measuring the deflection of the probe beam, using a position-sensitive detector (PSD) or a charge-coupled device (CCD) camera, the PDS signal is acquired which is proportional to the electromagnetic radiation absorption of the sample^27,28^.

Three configurations for experimental setup of the BDS system have been used: (a) transverse configuration where the probe beam is parallel to the sample surface and is utilized for opaque solid samples, (b) collinear configuration (or transmission configuration) where the probe beam transmits through the sample while the pump and probe beams are parallel and (c) reflection configuration in which the deflection of the reflected probe beam is measured^29,30^.

The BDS system as a sensor, introduces several advantages. This system does not require any complicated equipment. The low cost diode lasers could be exploited for pump and probe beams. Also simple equipment could be utilized as detector (e.g. quadrant photo detectors (QPD) which are usually operated as position sensitive detectors). Simple sample preparation, low required amount of samples along with high sensitivity as well as comparable spectral, spatial, and temporal resolution are advantages of the BDS as a detecting system^20,23,31,32^. Moreover, being contactless and non-destructive, make this system into a potential candidate for toxic and critical detections. In addition, the background measurement is zero which results in minimal calibration requirements^26^. The BDS system is humidity and temperature independent and could operate at high temperatures as well.

Various nanostructures have been extensively used to improve the selectivity and sensitivity of the H_2_S sensors^33–35^. For instance, detection of low concentrations of H_2_S has been performed using BaTiO_2_ nanoparticles^36^. Electroactive nanoparticles (NPs), such as metal NPs, have been utilized as electrode modifiers in electrochemical sensors in order to improve the generation of stable and strong electrochemical signals^2^. Among the various types of NPs which can be used in H_2_S detection, gold NPs have attracted considerable attention due to their favorable properties^37,38^. Biocompatible Au NPs have excellent conductivity, convenient functionalization properties and large specific surface area^39^, along with unique surface plasmon resonance (SPR) absorption peak at 524 nm^40^.

In comparison with other reducing (NH_3_) and oxidizing gases (Cl_2_ and NO_2_), the studies have demonstrated that Au NPs exhibited great selectivity for H_2_S molecules which could be attributed to the strong gold–thiol interaction^3,6,41–44^. Results of previous study showed that gold thin film reveal more selectivity toward H_2_S than NH_3_ and demonstrated a stronger response in similar condition. This phenomenon is due to the fact that H_2_S is more reducing in nature comparing with NH_3_^42^. On the other hand, due to the application of citrate in the process of synthesizing and stabilizing of gold nanoparticles, sulfide ions have high tendency to bind to gold nanoparticles and replace the carboxyl groups^45^. In this regard, using gold nanoparticles in the proposed sensing platform provides high selectivity for H_2_S detection. Furthermore, another study^40^, confirmed that the adsorption and desorption of H_2_S molecules led to significant changes in electron hopping of Au NPs which could be used in H_2_S detection. The morphology of gold nanostructures plays a critical role in their SPR absorption peaks. Spherical Au NPs show a single absorption peak while Au nano rods exhibit two peaks which are related to their longitudinal and transverse modes^46,47^. In SiO_2_–Au core–shell and Au–SiO_2_–Au nanomatryoshka structures, the peaks could be tuned with size of the core and shell structures accordingly^48,49^.

In this paper, we propose a novel H_2_S sensor structure based on the beam deflection technique and using Au nanostructures. The absorption spectrum of the Au nanostructures is dependent on the presence of gas in the medium. By irradiating the Au nanostructures-modified substrate, the generated heat would result in modifying the refractive index of the surrounding medium which could be detected by the deflection of the beam. Proposing the beam deflection approach makes the measurements easy (compared with other spectroscopic techniques such as SERS) while exploiting the Au NPs makes the sensing method selective and sensitive. Based on all optical methods exploited for this system, the functionality of sensing platform is independent of the humidity and temperature and unlike the electrochemical sensors, the operation of sensor would be possible in high temperatures as well. The system is easily operated and does not require complicated equipment. The operation of the system  is fundamentally based on the variation of electron density and the refractive index of Au nanostructures due to the adsorption of H_2_S molecules. Furthermore, exploiting the nanostructures provides a stronger response due to their high surface to volume ratio which provides higher surface for adsorption of H_2_S gas molecules. This improves the sensitivity of the proposed system. While the adsorption of H_2_S molecules on the surface of Au nanostructures is physical and no chemical reaction takes place, the recovery time of sensor is short. The performance of the proposed technique was computationally evaluated.

## Results

### Absorption coefficient of gold nanostructures

**Figure 1:**
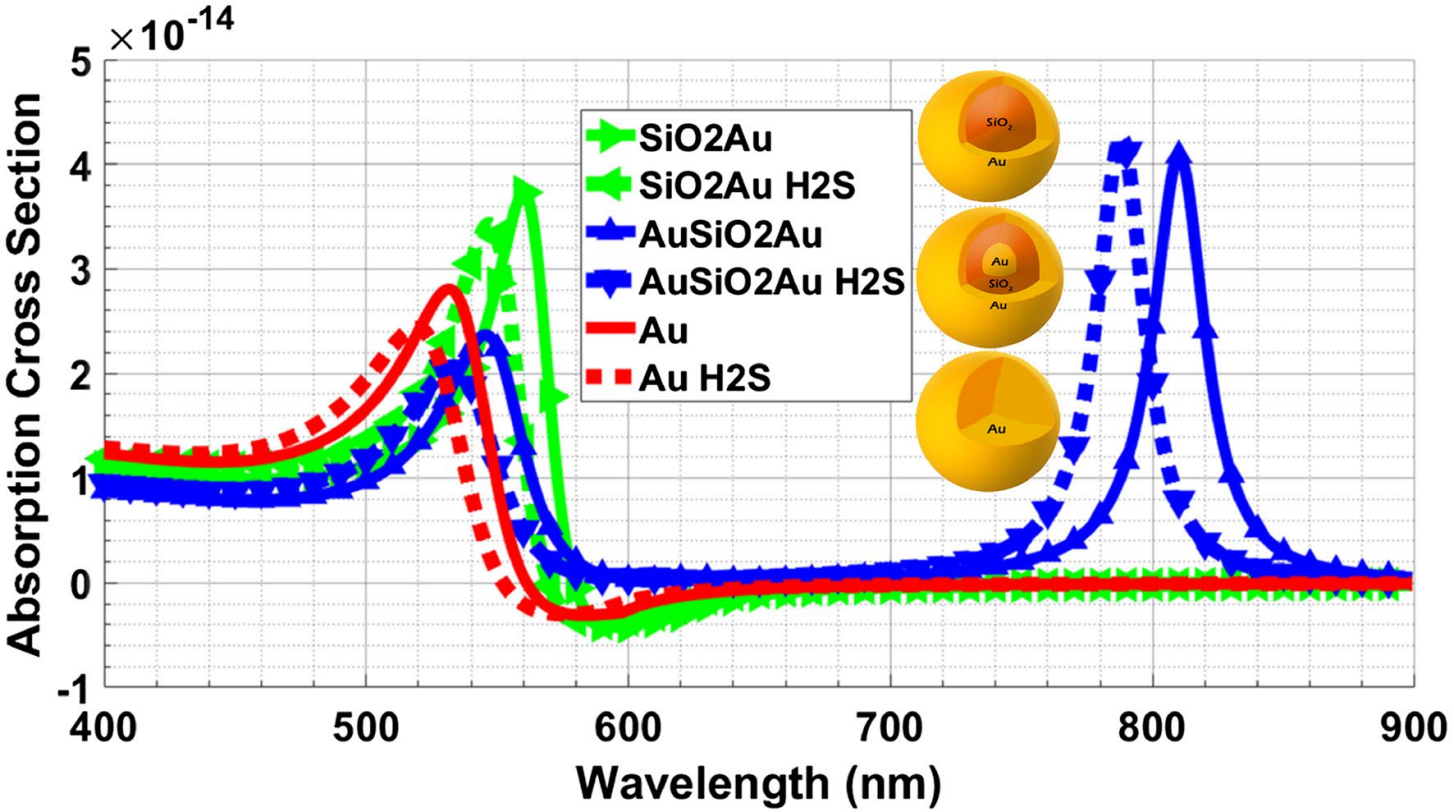
Absorption cross section of Au NPs.

The absorption cross section of three Au nanostructures calculated by solving Maxwell’s equations using the Mie method are illustrated in Fig. 1.

### Optimization of substrate

**Figure 2:**
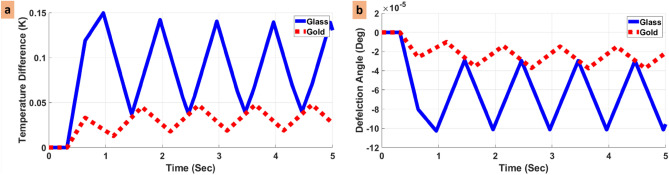
(**a**) Temperature difference and (**b**) deflection angle for the glass and Au substrates in the vicinity of H_2_S.

In order to find the appropriate substrate for the system, we compared the temperature variation and deflection angle for the modified-glass and modified-Au substrates in the vicinity of H_2_S. In this regard, Fig. 2 depicts the results of this comparison.

### Temperature variation profile of geometry

**Figure 3:**
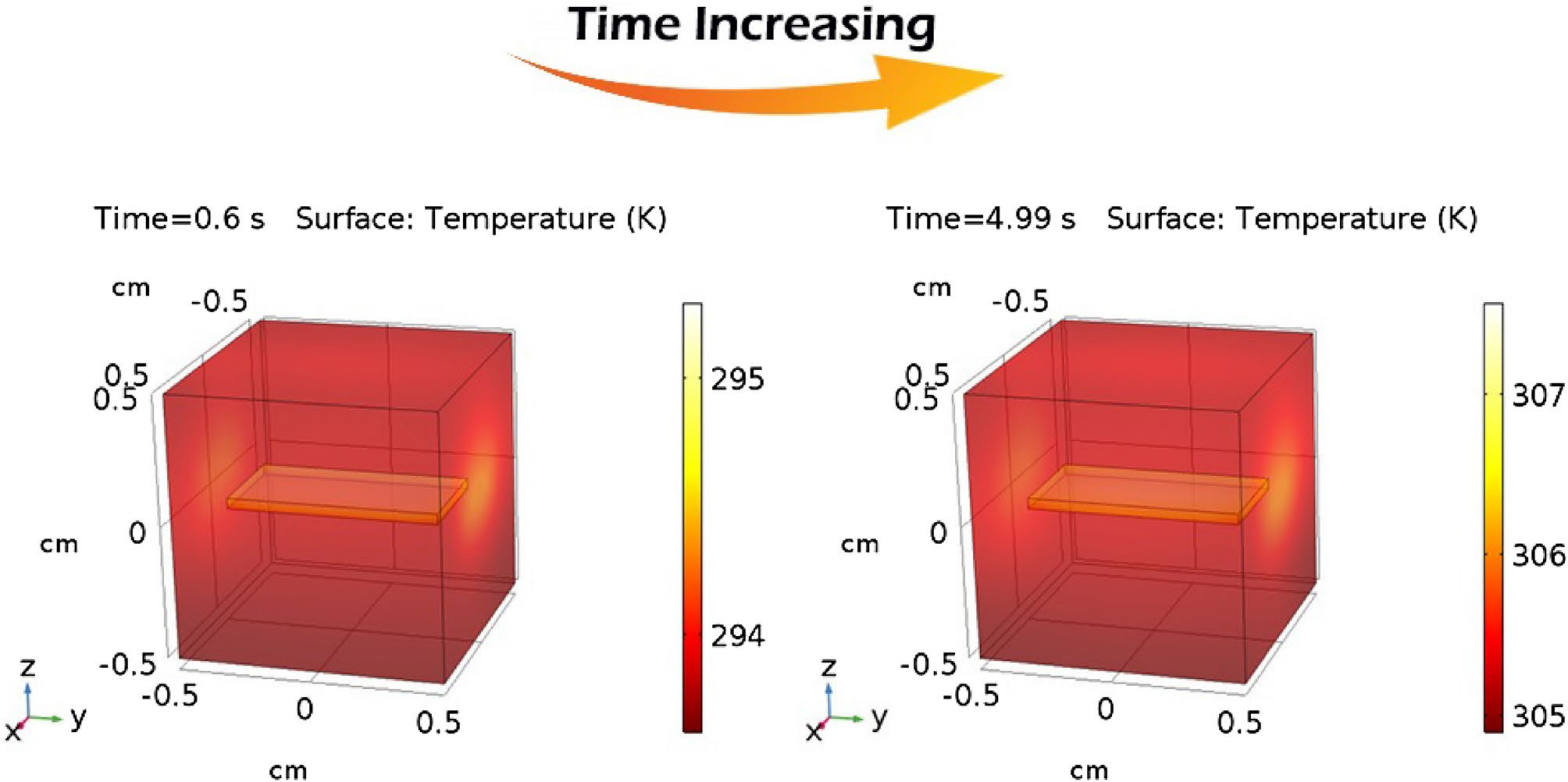
Temperature profile of geometry at two different simulation times.

Figure 3 demonstrates the temperature variation profile of geometry in the time domain at time intervals of 0.6 and 4.99 s, respectively.

### Temperature profile and deflection angle for Au nanostructures

**Figure 4:**
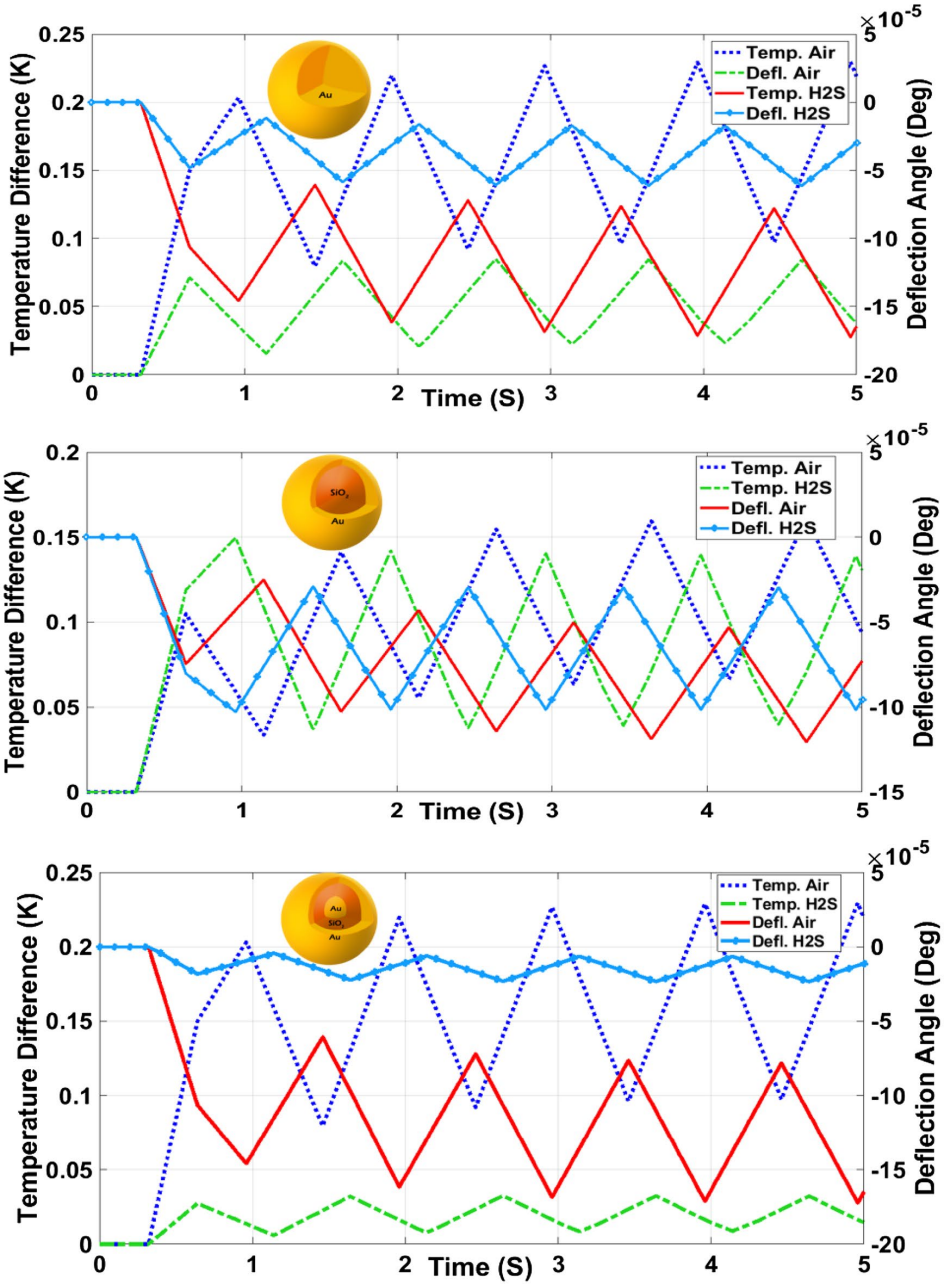
Temperature difference and deflection angle for (**a**) Au nanosphere, (**b**) SiO_2_–Au core–shell, and (**c**) Au–SiO_2_–Au nanomatryoshka in presence of air and H_2_S.

The BDS system with a modified substrate was used to detect air and H_2_S and the results were compared for three different Au nanostructures. Figure 4 shows the temperature variation and the deflection angle for these different nanostructures in the vicinity of air and H_2_S on the modified substrate.

## Discussion

We have proposed a BDS-based system for detection of H_2_S and its operational performance is simulated. Considering the application of Au nanostructures for improving the selectivity and sensitivity of detection, the absorption coefficient of three types of Au nanostructure (including Au nanosphere, SiO_2_–Au core–shell structure and Au–SiO_2_–Au nanomatryoshka structures) have been calculated. As shown in Fig. 1, the Au–SiO_2_–Au nanomatryoshka displays two absorption peaks compared with the two other types of Au nanostructures having one absorption peak which is based on the presence of Au in two separate regions of the nanostructure^50^. Moreover, a blue shift of the absorption peak for all three nanostructures in the vicinity of H_2_S is associated with an increase of electron density of Au nanostructure due to the adsorption of H_2_S molecules on the nanostructures^51^. As can be seen in Fig. 1, the blue shift for the Au–SiO_2_–Au nanomatryoshka is more distinctive than in the other two nanostructures.

Based on the important role of the substrate in transferring heat, we have compared the efficiency of the BDS for two different substrates. Figure 2 demonstrates the comparison of temperature variation and deflection angle for glass and Au substrates in the presence of H_2_S. As can be seen in Fig. 2, temperature variations and angle deflections for the glass substrate are more than those for the Au substrate. This difference is due to the fact that Au exhibits a higher heat conductance compared with the glass^52^. The smaller conductivity of the glass substrate would result in confining the heat near the surface of the substrate, while the Au substrate conducts the heat more easily, and the region between the Au and the gas achieves a lower heat. Considering the above reasons, the glass is a more appropriate substrate for the proposed BDS system.

Figure 3 demonstrates the temperature profile of the modelled geometry, showing the temperature raise in the structure in response to the absorbed heat from the pump-laser beam over time.

Finally, the efficiency of three different types of Au nanostructures on H_2_S detection by the BDS system was evaluated. Figure 4 shows the temperature variation and deflection angle of the laser beam in the vicinity of air and H_2_S for three different Au nanostructures. The results demonstrate that there are obvious differences in both the temperature variation and deflection angle between air and H_2_S for three types of Au nanostructures indicating the sensitivity of the proposed BDS system for detection of H_2_S. In addition, the difference is more pronounced for the Au–SiO_2_–Au nanomatryoshka structure, compared with the other two nanostructures. Thus, the maximum sensitivity of the BDS system for detection of H_2_S is through exploiting the Au–SiO_2_–Au nanomatryoshka. Using an Au nanostructure not only improves the selectivity of the system, but also introduces sensitivity through variation of electron density upon adsorption of the gas on its surface, which modifies its plasmon resonance behavior.

In our proposed modelling approach, the temperature dependency of thermal properties is negligible. For the high temperatures, this dependency should be considered and introduced in the heat transfer equation. In order to implement the system, for measurement and detection of the small variations of deflected beam, based on the periodical excitation of the sensitive layer by probe beam, the output signal could be detected with a lock-in approach. The lock-in detection could be performed either with an analog lock-in amplifier or digitally inside the software accordingly.

## Materials and methods

**Figure 5:**
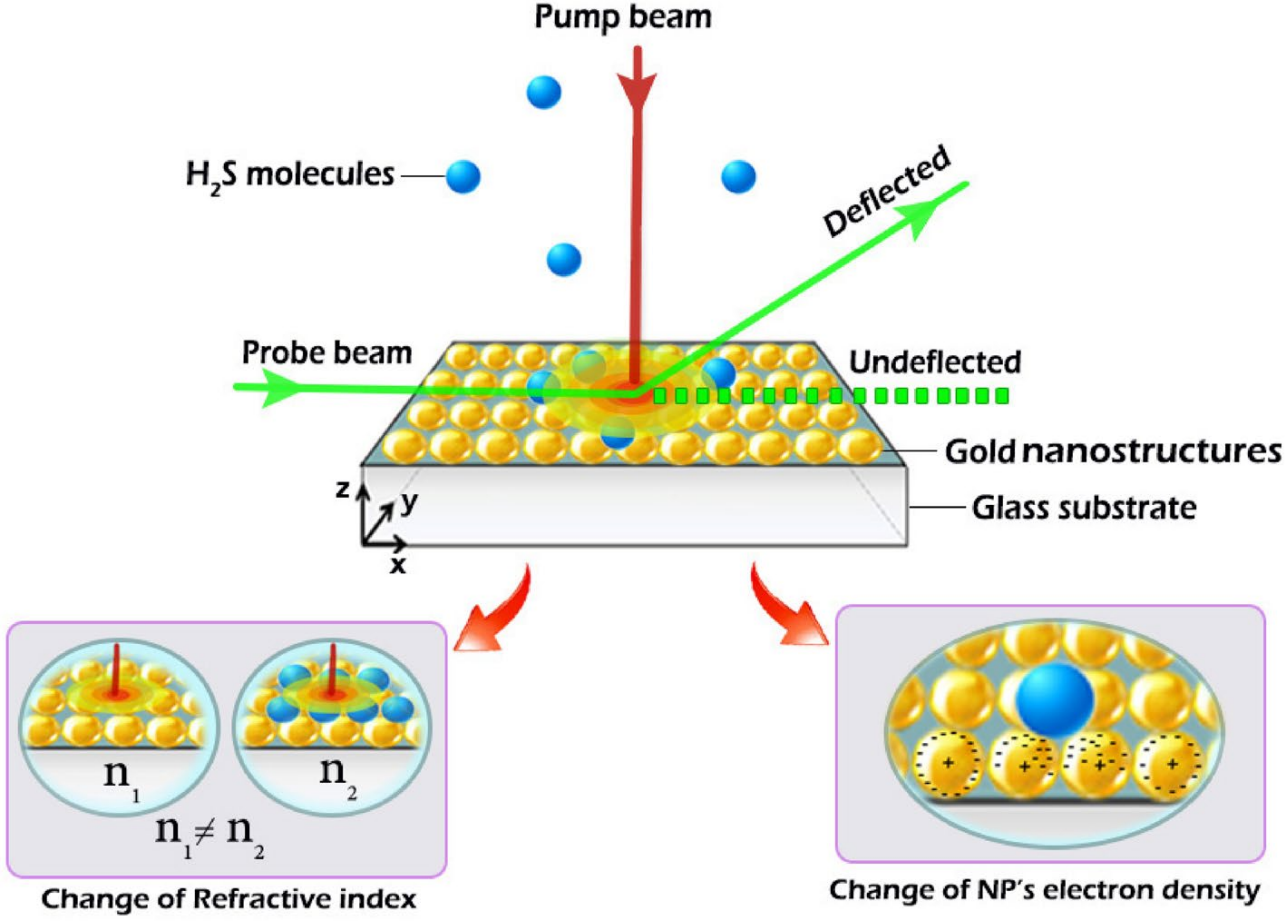
The schematic diagram of the proposed H_2_S detection system based on beam deflection.

As illustrated in Fig. 5, the sensing element of the proposed system is a substrate covered with Au nanostructures. The substrate is periodically heated with a laser beam (pump) passing through a chopper. The absorbed energy from the pump laser changes the refractive index of the adjacent medium. The modulation of the refractive index of the medium is detected through the deflection of a second laser beam (probe) which passes through the medium. The deflection could be detected by either the PSD or the CCD arrays. Based on the modulation of heat with the chopper, the deflection is modulated as well, which makes the detection easier using a lock-in amplifier.

Adsorption of H_2_S on the surface of Au nanostructures would result in the change in the electron concentration of the nanoparticles, which in turn leads to a change in the location of SPR peak and culminates in different values of the absorbed heat and deflection angle in the same manner. In order to model the functionality of the system, we have used computational modelling approach. First, the effect of gas adsorption on the absorption spectrum of three different Au nanostructures is modeled through solving Maxwell’s equations. The variation of the temperature in the surrounding medium of the sensing element (substrate + Au nanostructures) is calculated by solving the heat transfer equation via the finite element method (FEM) in the COMSOL Multiphysics version 5.3 environment^53^. Deflection of the laser beam resulting from the temperature gradient was calculated according to the governing equation for the propagation of laser beam.

### Optical properties of Au nanostructures in response to adsorbed gas

**Figure 6:**
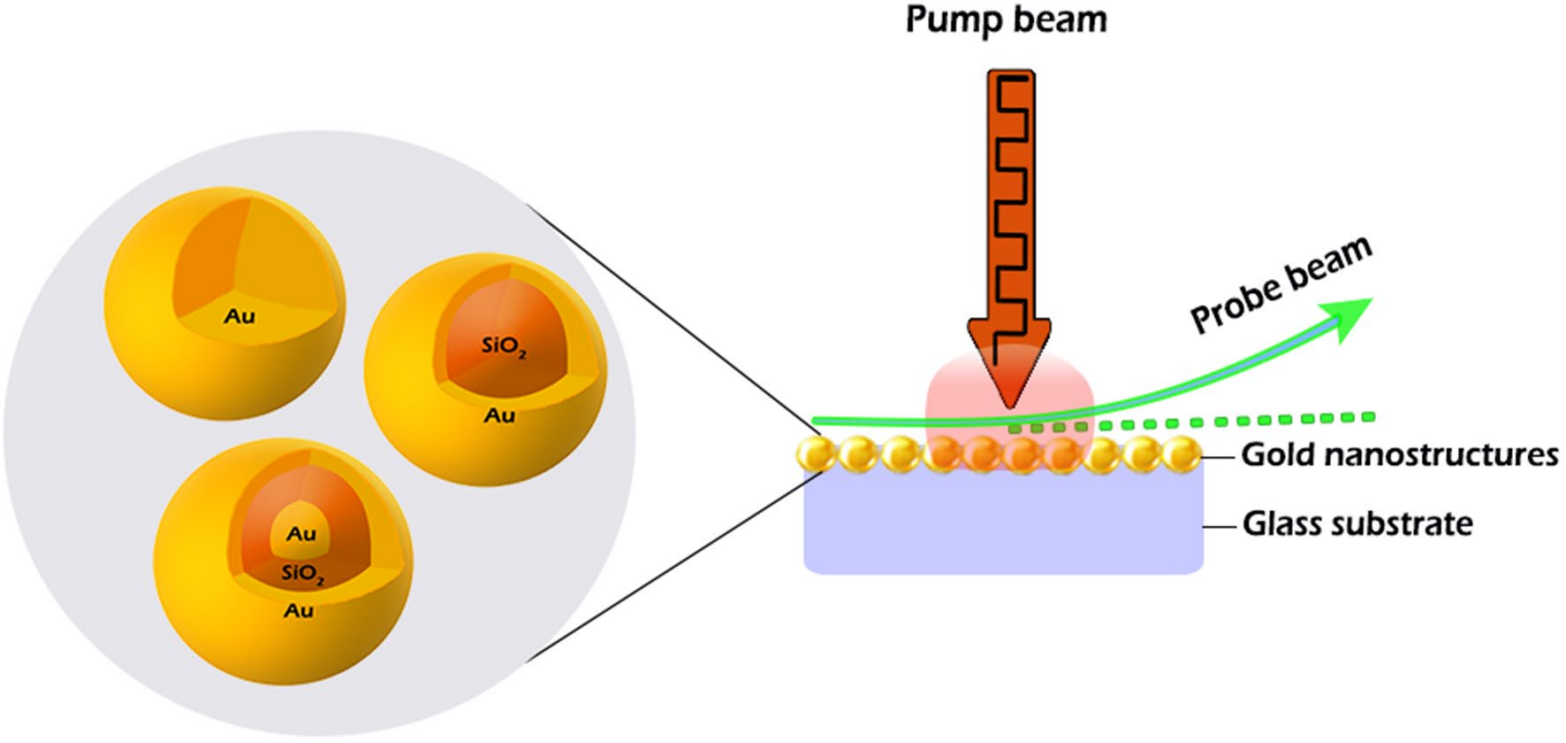
Different types of Au nanostructures; Au nanosphere, SiO_2_–Au core–shell and Au–SiO_2_–Au nanomatryoshka structures used for modifying the substrate.

Among various types of Au nanostructures with different geometries, as depicted in Fig. 6, we have selected three distinct structures including Au nanosphere, SiO_2_–Au core–shell and Au–SiO_2_–Au nanomatryoshka structures as the essential component of the sensing element. In order to find the optical properties of the above-mentioned nanostructures, Maxwell’s equations should be solved. Considering the spherical symmetry of all the three mentioned structures, Mie’s theory has been selected for solving Maxwell’s equations^54,55^. In this regard, the illuminated, scattered and absorbed waves are all expanded using spherical Bessel’s functions and imposing the boundary conditions for electric and magnetic fields on each boundary. The system of equations are solved to find the coefficients for each wave. Each structure and material is introduced in the equations by its permittivity and permeability. In order to introduce the dispersion behavior of the Au permittivity, various models have been introduced, among which we have used the Drude–Lorentz model

1 $$\varepsilon \left(\lambda \right)={\varepsilon }_{\infty }-\frac{1}{{\lambda }_{p}^{2}\left(\frac{1}{{\lambda }^{2}}+\frac{j}{{\gamma }_{p}\lambda }\right)}+\sum_{i=1}^{2}\frac{{A}_{i}}{{\lambda }_{i}}\left[\frac{{e}^{j{\varphi }_{i}}}{\left(\frac{1}{{\lambda }_{i}}-\frac{1}{\lambda }-\frac{j}{{\lambda }_{i}}\right)}+\frac{{e}^{-j{\varphi }_{i}}}{\left(\frac{1}{{\lambda }_{i}}+\frac{1}{\lambda }+\frac{j}{{\lambda }_{i}}\right)}\right]$$

to consider all the interband and intraband transitions^56^, where ɛ_∞_ is the dielectric constant far above the plasma frequency, λ_p_ denotes the plasma wavelength, ɣ_p_ is the damping factor expressed in wavelength. λ_i_ denotes the interband transition wavelength, ɣ_i_ is the transition broadenings (expressed as wavelength), A_i_ is the dimensionless critical point amplitude, and ɸ_i_ represents the phase.

### Effect of gas adsorption

The effect of gas adsorption on the permittivity and the variation of optical properties of Au nanostructures, in response to H_2_S adsorption, was taken into account to find the sensitivity of the proposed method. The plasma wavelength in Eq. (1) was calculated via

2 $${\lambda }_{p}=\sqrt{\frac{4{\pi }^{2}{c}^{2}m{\varepsilon }_{0}}{N{e}^{2}}}$$

where c is the velocity of light in vacuum, m represents the effective mass of the conduction electrons, ɛ_0_ is the vacuum permittivity, e is the electron charge, and N is the electron concentration. Adsorption of H_2_S on the surface of the Au nanoparticles, would locally increase the electron density which in turn reduces the plasma wavelength^51^.

### Temperature variation profile

**Figure 7:**
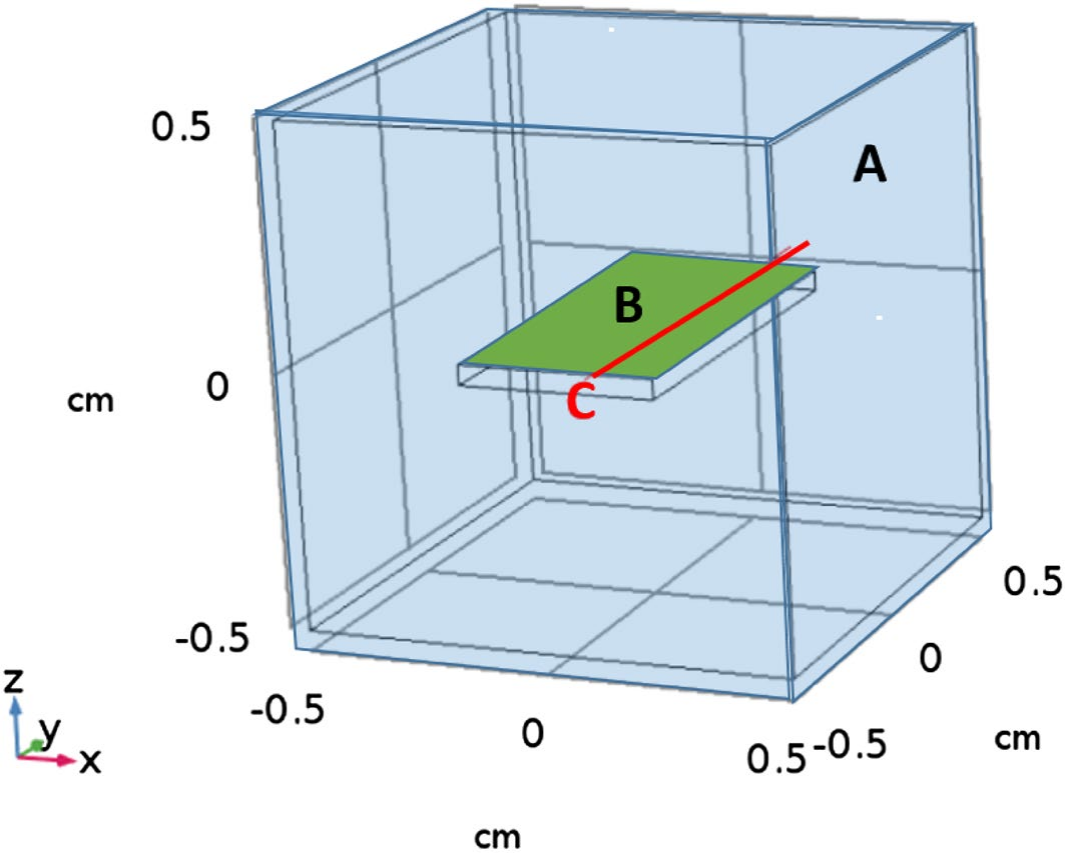
The geometrical structure of the model for the BDS system for detection of H_2_S gas. (A) Shows the H_2_S gas container (blue color), (B) shows the surface of substrate including the Au NPs (green color), and (C) demonstrates the cut line exploited for temperature variation (red color).

The main approach to calculate the beam deflection is to obtain the temperature variation profile during the heating of the sample. The temperature variations depend on the thermo-optical and structural features of the sample. In order to find the temperature variation profile, the heat transfer equation

3 $$\rho {\mathrm{C}}_{\mathrm{P}}\frac{\partial \mathrm{\delta T}}{\partial \mathrm{t}}+\left(-{\mathrm{K}\nabla }^{2}\delta T\right)=Q-\rho {\mathrm{C}}_{\mathrm{P}}u\cdot \nabla \mathrm{\delta T}$$

should be solved, where ρ is the density, C_p_ is the heat capacity, k is the thermal conductivity, u is the flow velocity, and Q represents the heat source^57,58^. In our model, the source of heat is the laser energy absorbed by the Au NPs. The wavelength-dependence absorption of various types of nanoparticles results in different values for the Q accordingly. The structure is heated with five consecutive Gaussian pulses with 1 second width. The geometrical structure of the model is depicted in Fig. 7. In order to solve Eq. (3) for the geometrical structure of Fig. 7, we used the FEM and the COMSOL Multiphysics environment. Equation (3) is solved in time domain and Table 1 lists the material parameters used in the model based on Eq. (3).

**Table 1:** Materials’ properties used in Eq. (3).

Material	Heat capacity [J kg^−1^ K^−1^]	Thermal conductivity [W m^−1^ K^−1^]	Density [kg^−1^ m^−3^]
Air	1020	0.0243	1.225
H_2_S	2240	0.14	1.5
Gold	219	314	19,300
Glass	840	0.8	2500

### Beam deflection calculation

After obtaining the temperature variation profile from the heat equation, the deflection of the probe-laser beam could be calculated. Generally, the time-dependent angle of deflection is calculated via

4 $$\theta \left(x,y,z,t\right)={\int }_{path}\frac{\partial {\delta }_{n}}{\partial {\delta s}_{\perp }}ds$$

where s denotes the path along which the probe-beam propagates in the x direction above the sample. Considering the deflection of the beam in the z direction, the angle of deflection can be calculated via

5 $$\theta \left(z,t\right)=\frac{dn}{dT}{\int }_{x}\frac{\partial \delta T\left(x,y,z,t\right)}{\partial z} dx$$

The value of dn/dT provides the variation of the refractive index with the temperature, and in this study, we have considered a fixed value of − 0.88 × 10^–6^ for this parameter^59^.

## Conclusion

A BDS-based system has been proposed for detection of H_2_S and its performance has been analyzed using computational modelling. The simulation results indicate that comparing Au and glass as a substrate, the glass is more appropriate due to its lower heat conductivity. Using Au NPs for modifying the substrate results in the selectivity of the system for H_2_S molecules due to the strong gold–thiol interaction. In addition, the plasmon resonance behavior of Au nanostructures would change due to the adsorption of gas on the surface of nanoparticles that alters the electron concentration locally. Among three proposed Au nanostructures, the Au–SiO_2_–Au nanomatryoshka exhibits a higher sensitivity for the detection of H_2_S. The proposed system has various advantages of rapid, reliable, sensitive and selective detection of the gas samples and could be employed in real-time applications.

## Data Availability

Derived data supporting the findings of this study are available from the corresponding author on request.
